# The S Genome Segment Is Sufficient to Maintain Pathogenicity in Intra-Clade Lassa Virus Reassortants in a Guinea Pig Model

**DOI:** 10.3389/fcimb.2018.00240

**Published:** 2018-07-11

**Authors:** Stephen R. Welch, Florine E. M. Scholte, César G. Albariño, Markus H. Kainulainen, JoAnn D. Coleman-McCray, Lisa Wiggleton Guerrero, Ayan K. Chakrabarti, John D. Klena, Stuart T. Nichol, Jessica R. Spengler, Christina F. Spiropoulou

**Affiliations:** Viral Special Pathogens Branch, Division of High-Consequence Pathogens and Pathology, Centers for Disease Control and Prevention, Atlanta, GA, United States

**Keywords:** Lassa virus, hemorrhagic fever, reassortment, guinea pig, reverse genetics, virus rescue

## Abstract

Genome reassortment in Lassa virus (LASV) has been reported in nature, but phenotypic consequences of this phenomenon are not well described. Here we characterize, both *in vitro* and *in vivo*, reassortment between 2 LASV strains: the prototypic 1976 Josiah strain and a more recently isolated 2015 Liberian strain. *In vitro* analysis showed that although cis- and trans-acting elements of viral RNA synthesis were compatible between strains, reassortants demonstrated different levels of viral replication. These differences were also apparent *in vivo*, as reassortants varied in pathogenicity in the guinea pig model of LASV infection. The reassortant variant containing the Josiah S segment retained the virulence of the parental Josiah strain, but the reassortant variant containing the S segment of the Liberian isolate was highly attenuated compared to both parental strains. Contrary to observations in reassortants between LASV and other arenavirus species, which suggest that L segment-encoded factors are responsible for virulence, these studies highlight a role for S segment-encoded virulence factors in disease, and also suggest that inefficient interactions between proteins of heterologous strains may limit the prevalence of reassortant LASV variants in nature.

## Introduction

Lassa virus (LASV, family *Arenaviridae*, genus *Mammarenavirus*) is the etiological agent of Lassa fever (LF), an acute zoonotic viral hemorrhagic fever endemic in West Africa. The first documented case occurred in 1969 in the area around Jos, Nigeria (Frame et al., 1970). The disease was soon recognized as being endemic in several West African countries, including Nigeria (Omilabu et al., 2005), Sierra Leone (Shaffer et al., 2014), Guinea (Kernéis et al., 2009), and Liberia (Frame et al., 1984; Monson et al., 1984). Recent data also indicate the presence of circulating strains in the surrounding countries of Mali (Safronetz et al., 2010, 2017), Ghana (Dzotsi et al., 2012), Benin (http://www.who.int/csr/don/13-june-2016-lassa-fever-benin/en/), Togo (Patassi et al., 2017; Whitmer et al., 2018), Burkina Faso (Frame, 1975; Swaan et al., 2002), and Côte d'Ivoire (Sogoba et al., 2012). Of the 100,000–300,000 estimated annual cases of LF, approximately 5,000 result in death (McCormick et al., 1987). Transmission most commonly occurs via direct contact with or inhalation of infectious excreta of *Mastomys natalensis*, the rodent vector. Person-to-person transmission can also occur though contact with infected body fluids, especially during outbreaks and in hospital settings (Fisher-Hoch et al., 1995; Lo Iacono et al., 2015).

**Figure 1:**
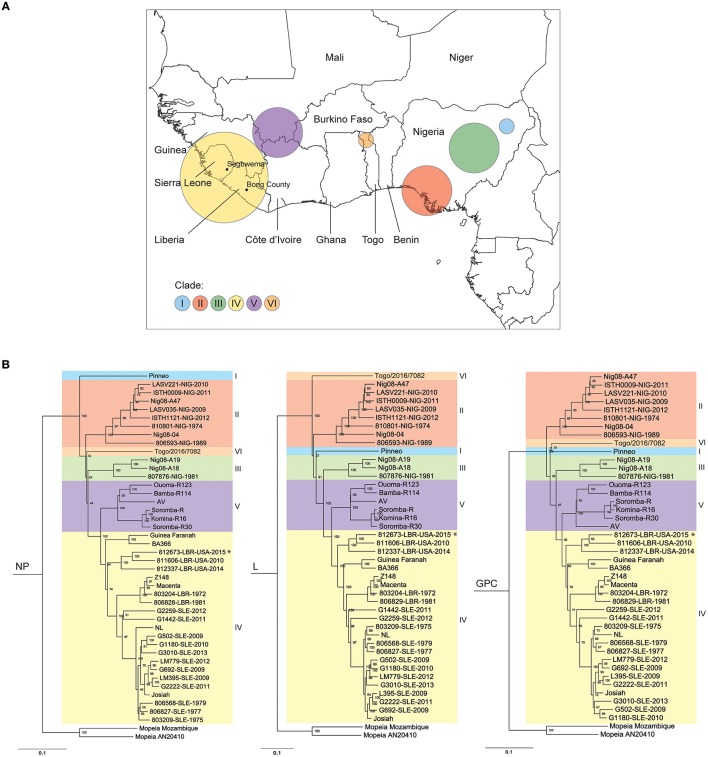
Distribution and phylogeny of Lassa virus strains in West Africa. **(A)** Sequence analysis of Lassa virus (LASV) strains demonstrates clustering based on geographical location, leading to the identification of 6 major lineages. Clades I–III are found in different regions of Nigeria; Clade IV in Sierra Leone, Liberia, and Guinea; Clade V in southern Mali; and Clade VI in Togo. Indicated are Segbwema, Sierra Leone, and Bong County, Liberia, from which LASV strain Josiah and 812673-LBR-USA-2015, respectively, originated. **(B)** Phylogenetic analysis of full-length nucleoprotein (NP), polymerase (L), and glycoprotein precursor (GPC) nucleotide sequences. Phylogenetic trees were rooted using 2 Mopeia virus (MOPV) isolates, and isolates were grouped by clades. LASV strain 812673-LBR-USA-2015 (NJ2015) indicated by ^*^. Node labels represent bootstrap values, 1,000 replicates. Scale bar represents 1 change per 100 nucleotides.

LASV has a bipartite, single-stranded ambisense RNA genome encoding 4 proteins: the nucleoprotein (NP) and glycoprotein precursor (GPC) on the small (S) segment, and the RNA-dependent RNA-polymerase (L) and matrix RING zinc-finger protein (Z) on the large (L) segment (Buchmeier et al., 2007). A striking feature of LASV is the high level of nucleotide diversity between strains, which can reach up to 32 and 25% for the L and S segments, respectively (Bowen et al., 2000; Ehichioya et al., 2011; Andersen et al., 2015). This diversity is correlated to geographic location, with clustering of strains leading to the recognition of 6 major LASV clades: I–III in Nigeria; IV covering the countries of Sierra Leone, Guinea, and Liberia; and V covering southern Mali. Another recently recognized clade, VI, originates from Togo (Figure 1A; Andersen et al., 2015; Whitmer et al., 2018).

Reassortment, the exchange of genome segments between different viral strains, occurs in a variety of segmented negative-sense RNA viruses (McDonald et al., 2016). LASV reassortment has been reported in nature (Andersen et al., 2015), but to our knowledge, no studies have investigated effects of reassortment on LASV phenotype. To do this, we used reverse genetics systems for 2 strains of LASV: a novel system based on strain 812673-LBR-USA-2015, isolated in 2015 from a fatal case of LF imported to New Jersey, USA, from Liberia; and a previously described system for LASV strain Josiah (Albariño et al., 2011). With these systems, we generated 2 reassortant viruses containing either an S or L genome segment from each parental strain. These 4 viruses were used to evaluate potential molecular barriers to LASV reassortment and characterize resultant recombinant variants.

## Materials and methods

### Cell culture

Vero-E6, BSR-T7/5, and A549 cells were cultured in Dulbecco's modified Eagle's medium (DMEM) supplemented with 10% (v/v) fetal calf serum, non-essential amino acids, 1 mM sodium pyruvate, 2 mM L-glutamine, 100 U/mL penicillin, and 100 μg/mL streptomycin. Huh7 cells were cultured in DMEM supplemented with 5% (v/v) fetal calf serum, non-essential amino acids, 100 U/mL penicillin, and 100 μg/mL streptomycin.

### Reverse genetics: viruses

LASV strain Josiah (henceforth called Josiah) is an isolate obtained from the serum of a 40-year-old male admitted to Songo hospital (Sebgwema, Sierra Leone) with LF in 1976. LASV strain 812673-LBR-USA-2015 (henceforth called NJ2015) is an isolate obtained from a 55-year-old male who succumbed to LF upon returning to New Jersey, USA, from Liberia in 2015. Infectious recombinant Josiah (rJosiah) was rescued as previously described (Albariño et al., 2011). Similarly, we generated recombinant NJ2015 (rNJ2015) by cloning full-length antigenomic sense copies of the NJ2015 S and L genome segments into T7 transcription plasmids. To create the reassortant recombinant rJos-S/NJ-L and rNJ-S/Jos-L variants, we alternated the S and L segment rescue plasmids used. All recombinant viruses were rescued in BSR-T7/5 cells and passaged twice in Vero-E6 cells. Viral titers were determined in Vero-E6 cells by immunofluorescence assays using anti-LASV polyclonal antibodies (in house, #SPR628), with TCID_50_ values calculated using the method of Reed and Muench (Reed and Muench, 1938). Sequences for rJosiah (HQ688673.1, HQ688675.1) and rNJ2015 (MG 812650, MG812651) have been deposited in Genbank.

### Reverse genetics: minigenome systems

Josiah S segment minigenome assays were performed, as previously described, by replacing the NP and GPC coding sequences (CDS) on the Josiah S segment with those for ZsGreen1 (ZsG) and *Gaussia* luciferase (gLuc), respectively (Welch et al., 2016). A new Josiah L segment-based minigenome was generated, replacing the L and Z CDS with those for ZsG and gLuc, respectively. Analogous NJ2015 S and L segment-based minigenomes and support plasmids expressing NJ2015 NP and L were also created. The 5′ and 3′ non-coding regions (NCR) and the intergenic regions were unaltered in all constructs. Cells transfected with minigenome segments and support plasmids supplying LASV NP and L proteins produced quantifiable ZsG and gLuc expression. All minigenome reactions were performed in conjunction with a negative control (no polymerase, just pCAGGS empty plasmid) to assess background levels of minigenome activity in the absence of viral transcriptional machinery. ZsG fluorescence or gLuc expression (Renilla Luciferase Assay System, Promega) was quantified 72 or 48 h post-transfection, respectively, on a BioTek Synergy microplate reader.

### Phylogenetics and sequencing

LASV sequences were obtained from both NCBI Genbank and isolates contained in the Centers for Disease Control and Prevention (CDC) virus collection. Sequencing was performed using TrueSeq reagents and analyzed on the MiniSeq system (both Illumina). Phylogenetic trees were constructed (neighbor-joining method, Jukes-Cantor nucleotide distance measure; bootstrap analysis based on 1,000 replicates) in CLC Genomics Workbench 9. Trees were visualized using FigTree v1.4.3 (University of Edinburgh, http://tree.bio.ed.ac.uk) and rooted using 2 Mopeia virus (MOPV) strains: Mozambique (Genbank DQ328874.1, DQ328875.1) and AN20410 (Genbank AY772170.1, AY772169.1).

### Western blot analysis

Protein lysates were harvested in 2 × Laemmli sample buffer and γ-irradiated (5 × 10^6^ rads). Proteins were denatured for 10 min at 95°C, separated on 4–12% Bis-Tris SDS-PAGE gels, and transferred to nitrocellulose membranes using a semi-dry blotting system (Bio-Rad). LASV NP and GPC/GP1 were detected with mouse monoclonal antibodies generated in-house. Tubulin, used as a loading control, was detected with anti-tubulin antibody at 1:10,000 (Sigma, #T5168). Cellular proteins were detected with anti-STAT1 at 1:500 (BD BioScience, #610120), anti-pSTAT1 at 1:500 (Cell Signaling, #9171), and anti-ISG15 at 1:1000 (ProteinTech, #15981-1-AP) antibodies. Secondary antibodies were detected with Supersignal West Dura Fast Western Blot kits (Thermo Fisher Scientific) and visualized using a Bio-Rad ChemiDoc MP imaging system.

### Ethics statement

All animal procedures were approved by the CDC Institutional Animal Care and Use Committee (#2833SPEGUIC) and conducted in accordance with the *Guide for the Care and Use of Laboratory Animals* (National Research Council of the National Academies, 2011). The CDC is fully accredited by the Association for Assessment and Accreditation of Laboratory Animal Care International. Procedures conducted with LASV or LASV-infected animals were performed in the CDC biosafety level 4 laboratory.

### Guinea pig infections

A total of 22 strain 13/N guinea pigs (males and females aged 6 months to <4 years) were obtained from our breeding colony at the CDC (Atlanta, GA). Groups of 5–6 age- and sex-matched guinea pigs were inoculated subcutaneously with 1 × 10^4^ focus-forming units (FFU; equivalent to ~2 × 10^4^ TCID_50_) of rJosiah (GenBank: HQ688673.1, HQ688675.1), rNJ2015 (GenBank: MG812679, MG812678), rJos-S/NJ-L, or rNJ-S/Jos-L. FFU, and TCID_50_ titers were calculated in Vero-E6 cells by immunofluorescence assays using anti-LASV polyclonal antibodies, with the latter calculated using the method of Reed and Muench (Reed and Muench, 1938). All animals were housed individually on deep soft bedding and given daily fresh vegetable enrichment and commercial guinea pig chow and water *ad libitum*. Animals' health was assessed by experienced CDC veterinarians or animal health technicians. Animals were humanely euthanized with isoflurane vapors and sodium pentobarbital (SomnaSol Euthanasia-III solution; Henry Schein Animal Health) once clinical illness scores (including, but not limited to, piloerection, ocular discharge, weight loss >25% of baseline at −1 dpi, changes in mentation, ataxia, dehydration, dyspnea, and/or hypothermia) indicated that the animal was in the terminal stages of disease, or at the completion of study 41 days post-infection (dpi).

### Quantitative real-time PCR

RNA was extracted from blood and homogenized tissue samples using the MagMAX-96 Total RNA Isolation Kit (Thermo Fisher Scientific) on a 96-well ABI MagMAX extraction platform with a DNase-I treatment step according to manufacturer's instructions. RNA was quantified by a qRT-PCR assay targeting a strain-specific NP gene sequence (primer and probe sequences available on request) and normalized to 18S RNA levels. Viral S segment copy numbers were determined using standards prepared from *in vitro*-transcribed S segment RNA. Levels of IFN-β, IFN-λ1, ISG56, CCL5, and IL-6 transcripts in infected A549 cells were determined using commercial assays (all Thermo Fisher, #4331182).

### Serology

Plasma was separated from EDTA-blood by centrifugation at 6,000 rcf and γ-irradiated (5 × 10^6^ rads). Samples were diluted 1:25 in master plate diluent (5% skim milk powder, 0.5% Tween-20, 0.5% Triton X100, and 0.01% thimerosal in PBS). To determine anti-LASV IgG levels, background was first reduced by pre-absorption on plates coated with Vero-E6 lysate. The samples were then further diluted as a 4-fold dilution series (1:100–1:6,400) in 5% skim milk powder and 0.1% Tween-20 in PBS, and incubated for 1 h at 37°C on plates coated with either Vero-E6 lysates or lysates from Josiah-infected Vero-E6 cells. After washing with 0.1% Tween-20 in PBS, bound guinea pig IgG was detected by incubation with an HRP-conjugated secondary antibody for 1 h at 37°C (KPL), washing, and adding ABTS Microwell Peroxidase Substrate (KPL) for 30 min at 37°C to develop absorbance signals. The raw data were first corrected by subtracting the value obtained with unspecific lysates (Vero-E6) from the specific signal (lysates from infected cells). Samples were considered positive for anti-LASV IgG if: (1) individual well signals were >2 standard deviations higher than the average of known negatives; and (2) summed signals from all dilutions for that sample were >2 standard deviations above the average of the known negatives. When 2 repeated measurements gave a positive signal for a sample, the average titer was reported. Otherwise the sample was reported as negative. Total IgM were captured by goat anti-guinea pig IgM antibody (ICL Lab, #GM-60A) and incubated with cell slurries from mock-infected or LASV-infected Vero-E6 cells, and anti-LASV IgM were detected using a polyclonal antibody against LASV (in-house reagent, HMAF #703079) and secondary anti-mouse IgG HRP-conjugate (Pierce, #31446). IgM results were analyzed like IgG results, except that 3 standard deviations were used to determine the cut-off for positive results.

### Clinical chemistry

Whole blood samples collected in lithium heparin were analyzed on Piccolo Xpress chemistry analyzers (General Chemistry 13 Panel, Abaxis) within 1 h of collection. Normal values were determined based on samples obtained from in-house colony of strain 13/N guinea pigs aged 6 months to <4 years (*n* = 101). Range was calculated at mean ±1 standard deviation.

### Statistical analyses

Minigenome and innate immune response qRT-PCR data were analyzed by one-way ANOVA with Dunnett's multiple comparison test. Survival was analyzed by Log-Rank (Mantel-Cox) test. All analyses were performed using GraphPad Prism v7.0 software.

## Results

### NJ2015 clusters in clade IV with other liberia LASV isolates, but remains genetically distinct from josiah strain

Complete nucleotide CDS of NJ2015 NP, L, and GPC were aligned with those of both historical and recent LASV isolates (Supplementary Table 1). NJ2015 sequences clustered closely with other Liberian isolates within clade IV, but remained distinct from both Josiah and other more recently isolated Sierra Leone strains (Figure 1B). Nucleotide lengths of the 4 CDS were identical between NJ2015 and Josiah, but NCR and intragenic region lengths in both genome segments varied (Table 1). The most striking difference was the minimal L segment 5′-NCR of NJ2015 (52 nt) compared to Josiah (157 nt). Diversity was greatest in the L segment CDS, demonstrating 79.3% (L) and 79.9% (Z) nucleotide identity. Nucleotide identity was 82.6% for NP and 83.7% for GPC (Table 2). Amino acid identity was higher than nucleotide identity in all 4 proteins, ranging from 87.7% (L) to 96.4% (GPC).

**Table 1:** Comparison of the coding and non-coding regions of Josiah and NJ2015 LASV strains (values are nucleotide length).

	**S Genome segment**					**L Genome segment**				
	**5′ NCR**	**NP**	**IGR**	**GPC**	**3′ NCR**	**5′ NCR**	**L**	**IGR**	**Z**	**3′ NCR**
Josiah	100	1,710	61	1,476	54	157	6,663	100	300	65
NJ2015	94	1,710	58	1,476	52	52	6,663	99	300	65

*NCR, non-coding region; IGR, intergenic region; L, RNA-dependent RNA polymerase; Z, RING finger protein Z; NP, nucleoprotein; GPC, glycoprotein precursor*.

**Table 2:** Nucleotide and amino acid homology between LASV strains.

	**Josiah vs. NJ2015**		Josiah vs. modern SL strains^*^		Modern SL strains^*^ **vs. modern LBR strains**^†^	
	**nt**	**aa**	**nt**	**aa**	**nt**	**aa**
Nucleoprotein	82.6	94.4	94.2 (88.0–96.5)	97.8 (95.8–99.0)	82.9 (81.4–84.3)	94.3 (92.8–95.3)
Glycoprotein precursor	83.7	96.4	93.9 (87.5–96.1)	98.3 (95.9–99.4)	83.0 (81.2–85.2)	95.6 (93.7–97.4)
Polymerase	79.3	87.7	93.3 (85.1–96.2)	96.4 (92.1–98.2)	79.2 (78.1–80.0)	87.1 (85.9–87.9)
Z	79.9	88.0	92.7 (83.7–96.3)	93.8 (87.0–98.0)	79.5 (74.3–84.2)	84.7 (75.0–89.0)

When comparing multiple strains, mean homology is represented, with range in parentheses (all values indicate % homology).

*nt, nucleotide; aa, amino acid; SL, Sierra Leone; LBR, Liberia*.

* *LASV strains isolated from Sierra Leone between 2009 and 2013 (Andersen et al., 2015)*.

† *LASV strains isolated from Liberia between 2010 and 2015*.

Temporal genetic variation, even within clades, was less evident. Although isolated in 1976, Josiah retains remarkable nucleotide and amino acid identity to modern Sierra Leonean strains (Andersen et al., 2015; Table 2). Unfortunately, no extensive database of recent Liberian LASV strains exists for comparison, although NJ2015 clustered closely with 2 recent Liberian isolates included in our analysis. These recent Sierra Leonean and Liberian strains share 79–83% nucleotide identity across all 4 CDS. Amino acid identity was greatest in S segment-encoded proteins (95% for both NP and GPC), and lower in L segment-encoded proteins (87% for L and 84% for Z).

### The Cis- and trans-acting elements required for viral RNA synthesis are interchangeable between josiah and NJ2015

**Figure 2:**
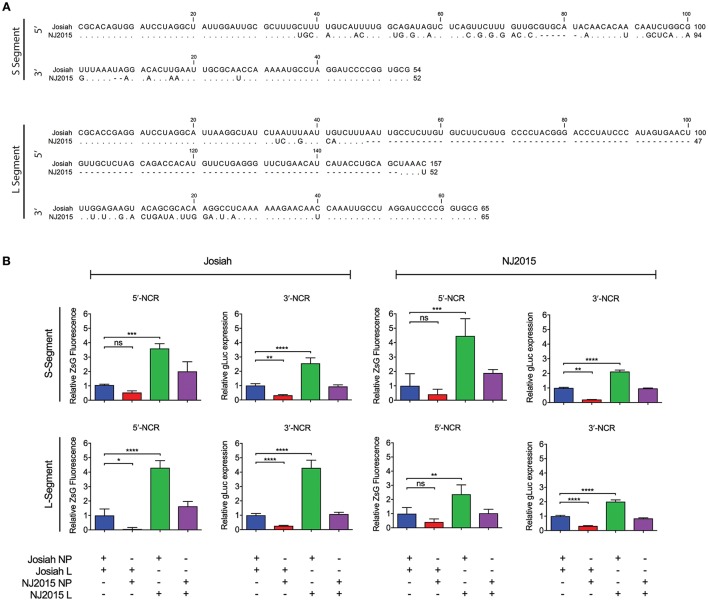
Minigenome analysis of LASV strains NJ2015 and Josiah. **(A)** Alignments of the 5′ and 3′ non-coding regions (NCRs) of the S and L genome segments of Josiah and NJ2015. Sequences are represented in the antigenomic viral complementary sense, with matching nucleotides shown as dots (.). (**B)** Minigenomes were designed to quantify relative transcriptional strengths of the 5′ NCR (measure by ZsG expression) or 3′ NCR (measured by gLuc expression) on the S and L genome segments of both Josiah and NJ2015 strains of LASV. The NP and GPC open reading frames (ORFs) (S segment), and the L and Z ORFs (L segment) were replaced by the ZsGreen (ZsG) and *Gaussia* luciferase (gLuc) ORFs, respectively. Huh7 cells were transfected with minigenome segments in conjunction with NP and L from either of the 2 LASV strains. Total ZsG fluorescence or gLuc expression was determined at 48 h post-transfection, with values represented normalized to Josiah-based minigenome segment with Josiah NP and Josiah L. Bar represents the mean of quadruplicate wells, with error bars indicating standard deviation; graphs are representative of 3 independent experiments. Lines represent statistical significance based on one-way ANOVA with Dunnett's multiple comparison test (^*^p < 0.05; ^**^p < 0.01; ^***^p < 0.001; ^****^p < 0.0001; ns, not significant).

Arenavirus NCRs contain highly conserved 19–25 terminal nucleotides responsible for maintaining the panhandle structure of arenavirus genomes, and cis-acting promoter elements for viral RNA (vRNA) synthesis (Buchmeier et al., 2007). Aligning Josiah and NJ2015 NCRs demonstrated this conservation of the terminal nucleotide sequences. Both S and L segment NCRs varied immediately upstream of the start codon (Figure 2A). The NJ2015 L segment 5′ NCR was 67% shorter than the Josiah equivalent, but retained 32 conserved nucleotides at the terminus. Using minigenome assays based on Josiah and NJ2015 genome segments, we evaluated the relative activities of both the cis-acting (NCRs) and trans-acting elements (NP and L) required for vRNA synthesis using cognate and non-cognate combinations. Minigenome reporter gene activity values are presented as relative to activity values determined using Josiah NP and L (Figure 2B; raw values for ZsG fluorescence and gLuc luminescence in Supplementary Figure 1). The activity of both NJ2015 S and L segment-based minigenomes with cognate NP and L was comparable to activity of both Josiah S and L segment-based minigenomes with cognate proteins. When Josiah NP and NJ2015 L were used in conjunction, minigenome activity significantly increased 2- to 5-fold over activity of cognate NP and L, independently of NCR origin. Conversely, minigenome activity significantly declined when NJ2015 NP was used together with Josiah L, again independently of NCR origin. These results show that the cis-acting and trans-acting elements of these LASV strains are interchangeable, although minigenome activity changes depending on the combination of NP and L used.

### LASV intra-clade reassortant viruses can be generated by reverse genetics

**Figure 3:**
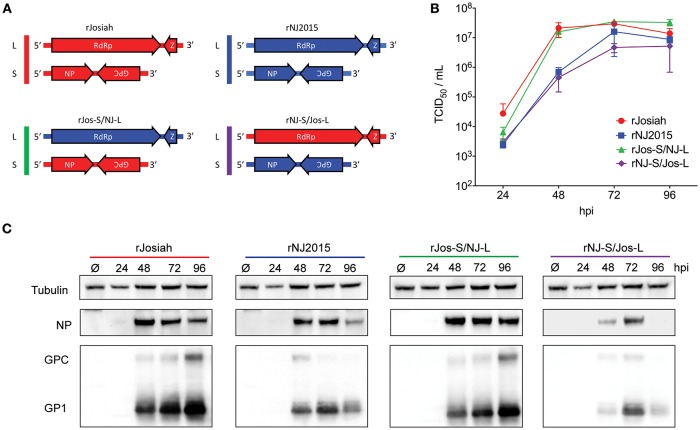
Characterization of NJ2015 and 2 strains produced by reassortment of NJ2015 and Josiah. **(A)** Schematic representation of the genome arrangements of the parental recombinant Josiah (rJosiah) and rNJ2015 strains, as well as the 2 reassortant variants rJos-S/NJ-L and rNJ-S/Jos-L (RdRp, RNA-dependent RNA polymerase; Z, matrix RING zinc-finger protein Z; NP, nucleoprotein; GPC, glycoprotein precursor) and **(B)** comparison of their growth kinetics in A549 cells infected at MOI 0.1. Virus titers (TCID_50_) were determined at 24, 48, 72, and 96 h post-infection (hpi). **(C)** S segment-encoded viral protein expression levels in in A549 cells infected at MOI 0.1. Cell lysates were harvested 24, 48, 72, and 96 hpi to determine cellular levels of NP and GPC and its cleavage product GP1. Uninfected A549 cells harvested 96 hpi were used as mock (Ø), and tubulin was used as loading control.

A reverse genetics system to rescue infectious recombinant Josiah (rJosiah) from DNA plasmids has been described previously (Albariño et al., 2011). We created an analogous system to rescue NJ2015 (rNJ2015), cloning full-length antigenomic-sense S and L genome segments into transcription plasmids under control of a T7 polymerase promoter to rescue virus in BSR-T7/5 cells (Figure 3A). By alternating rescue plasmids, we also rescued 2 reassortant variants: one containing the Josiah S segment and NJ2015 L segment (rJos-S/NJ-L), and the other containing NJ2015 S segment and Josiah L segment (rNJ-S/Jos-L). rJosiah and rJos-S/NJ-L showed similar growth kinetics in A549 cells, reaching maximum titers at 48 hpi (Figure 3B). rNJ2015 and rNJ-S/Jos-L exhibited an attenuated phenotype compared to rJosiah and rJos-S/NJ-L, with lower titers at 24 and 48 hpi, and approximately 10-fold lower end-point titers. Successful rescue and propagation of both rJos-S/NJ-L and rNJ-S/Jos-L viruses demonstrated that reassortment between Josiah and NJ2015 genome segments can result in viable virus variants.

Reassortment altered viral protein expression profiles of S segment-encoded proteins (Figure 3C). NP levels in cells infected with rJosiah and rNJ2015 were first detectable 48 hpi and persisted until 96 hpi. Expression levels were equivalent between strains at 48 and 72 hpi, but rNJ2015 levels dropped slightly at 96 hpi. Levels of GPC and the associated cleavage product GP1 were slightly higher in rJosiah-infected cells than in rNJ2015-infected cells, with levels in the latter decreasing over time. Protein expression levels were similar in rJos-S/NJ-L-infected and rJosiah-infected cells, although the former expressed slightly more NP. The opposite was true of rNJ-S/Jos-L infected cells, with expression of both proteins appearing later and at lower levels than in NJ2015-infected cells. LASV NP and Z proteins are known to antagonize various aspects of the immune system (Martínez-Sobrido et al., 2007; Qi et al., 2010; Hastie et al., 2012; Pythoud et al., 2012; Rodrigo et al., 2012; Jiang et al., 2013; Huang et al., 2015; Xing et al., 2015). However, transcription levels of IFN-β, IFN-λ, ISG56, CCL5, and IL-6, and expression levels of STAT1, pSTAT1, and ISG15 were similar in A549 cells after infection with all 4 recombinant viruses (Supplementary Figure 2). Therefore, although viral protein expression was altered in the reassortants, these differences did not appear to result in differences in immune suppression.

### S segment of josiah is sufficient to retain virulence in guinea pig model

**Figure 4:**
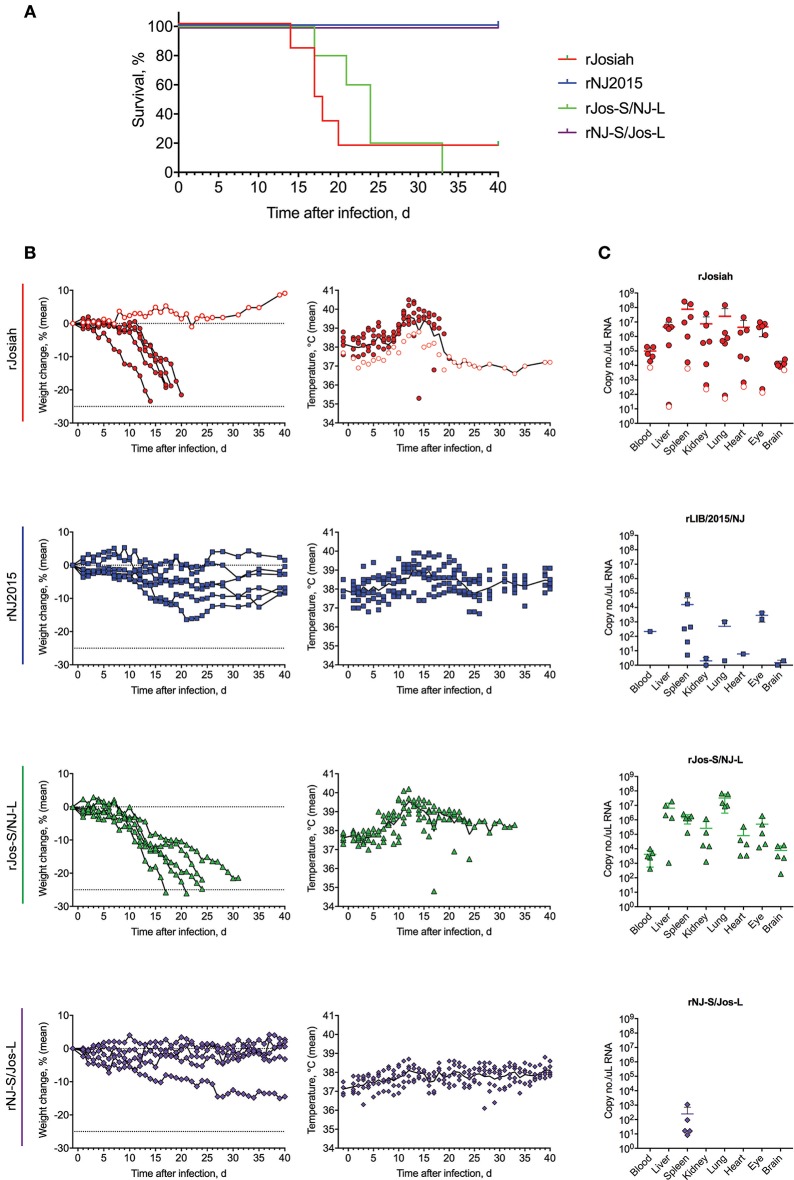
Survival, clinical data, and terminal viral loads in tissues of guinea pigs infected with LASV variants. **(A)** Survival curve of 13/N guinea pigs inoculated subcutaneously with 1 × 10^4^ FFU of rJosiah (*n* = 6), rNJ2015 (*n* = 6), rJos-S/NJ-L (*n* = 5), or rNJ-S/Jos-L (*n* = 5). **(B)**. Daily temperature and weight loss data from the infected guinea pigs. The white circle with red border represents the surviving rJosiah-infected animal. Temperature data for individual animals are shown, with the line representing the mean. **(C)** LASV loads in select tissue samples determined by qRT-PCR. Individual values are represented, with means and standard deviation shown. The white circle with red border represents the surviving rJosiah-infected animal.

**Figure 5:**
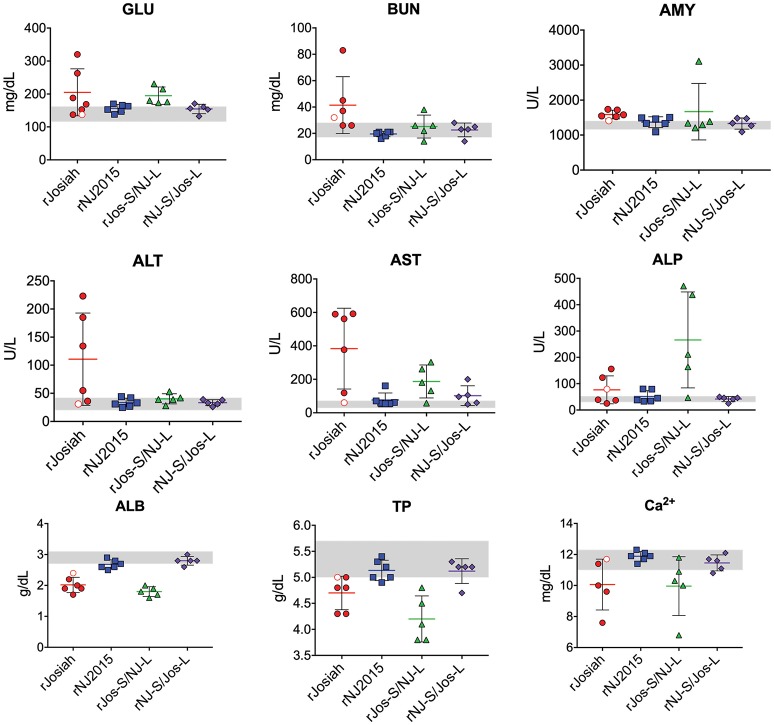
Clinical chemistry values in guinea pigs infected with LASV variants. Clinical chemistry was performed on whole blood samples collected at the time of euthanasia. Individual values are represented, with means and standard deviation shown. The white circle/red border represents the surviving rJosiah-infected animal. Gray shading indicates the normal range of analytes in colony animals aged 6 months to 4 years (*n* = 101, mean ± 1 SD). GLU, glucose; BUN, blood urea nitrogen; Ca^2+^, calcium; ALB, albumin; TP, total protein; ALT, alanine aminotransferase; AST, aspartate aminotransferase; ALP, alkaline phosphatase; AMY, amylase.

To investigate how phenotypic differences observed *in vitro* translate *in vivo*, we inoculated strain 13/N guinea pigs with 1 × 10^4^ FFU of rJosiah, rNJ2015, rJos-S/NJ-L, or rNJ-S/Jos-L. Similar to previous reports, animals inoculated with rJosiah succumbed to disease 14–20 dpi (Jahrling et al., 1982; Carrion et al., 2007; Cashman et al., 2011, 2013); one animal did not display overt clinical signs and was euthanized at study completion (41 dpi) (Figure 4A). Animals inoculated with rNJ2015 exhibited weight loss and elevated temperatures, but all recovered and were euthanized at study completion (Figure 4B). The clinical course of animals inoculated with the reassortants largely mirrored that of the parental viral strain from which the S segment originated. Animals inoculated with rJos-S/NJ-L animals exhibited acute weight loss beginning on 10 dpi, and all succumbed to disease 17–24 dpi. In contrast, most rNJ-S/Jos-L-inoculated animals exhibited no observable progressive weight loss or elevated temperatures, and all survived until study completion (41 dpi). Similarly, vRNA was widely disseminated and detected at high levels in both blood and tissues of animals with terminal disease upon infection with either rJosiah or rJos-S/NJ-L (Figure 4C). Low or no vRNA levels were detected at study completion in animals infected with rNJ2015, and vRNA was detected only in the spleens of rNJ-S/Jos-L-infected animals. Clinical chemistry analyte values were comparable between rJosiah- and rJos-S/NJ-L-infected animals, and between rNJ2015- and rNJ-S/Jos-L-infected animals (Figure 5). In animals that succumbed to rJosiah or rJos-S/NJ-L infection, analyte abnormalities included hypocalcemia, hypoalbuminemia, and decreased total protein; in rNJ2015- and NJ-S/Jos-L-infected survivors, these values were within normal limits at study completion. Compared to rNJ2015- and NJ-S/Jos-L-infected survivors, mean AST increased at least 3.75- and 1.83-fold in rJosiah- and rJos-S/NJ-L-infected animals, respectively; however, mean ALT was only higher in rJosiah-infected animals, but correlated to the relative magnitude of the more elevated AST levels seen in some of the animals. Anti-LASV NP IgG was detected in all surviving animals, but not in succumbing animals (Table 3). IgM was detected in 5 of 6 rJosiah-infected guinea pigs and in 1 of 5 rJos-S/NJ-L-infected guinea pigs.

**Table 3:** Plasma antibody levels in guinea pigs mock-infected or infected with one of 4 recombinant Lassa viruses.

**Sample**	**DPI**	ELISA^*^		**IFA**		
		**IgG**	**IgM**	**NP**	**GPC**	**Z**
Mock-1	N/A	0	0	–	–	–
Mock-2	N/A	0	0	–	–	–
Mock-3	N/A	0	0	–	–	–
Mock-4	N/A	0	0	–	–	–
Mock-5	N/A	0	0	–	–	–
rJosiah-1	14	0	400	–	–	–
rJosiah-2	41	6,400	850	+	+	–
rJosiah-3	20	0	400	+	–	–
rJosiah-4	17	0	1,000	+	–	–
rJosiah-5	17	0	0	+	–	–
rJosiah-6	18	0	250	+	–	–
rNJ2015-1	41	250	0	+	–	–
rNJ2015-2	41	1,600	0	+	–	–
rNJ2015-3	41	6,400	0	+	–	–
rNJ2015-4	41	6,400	0	+	–	–
rNJ2015-5	41	6,400	0	+	–	–
rNJ2015-6	41	6,400	0	+	+	–
rJos-S/NJ-L-1	24	0	0	+	–	–
rJos-S/NJ-L-2	17	0	0	+	–	–
rJos-S/NJ-L-3	24	0	0	+	–	–
rJos-S/NJ-L-4	33	0	0	+	–	–
rJos-S/NJ-L-5	21	0	4,000	+	–	–
rNJ-S/Jos-L-1	41	4,000	0	+	–	–
rNJ-S/Jos-L-2	41	1,600	0	+	+	–
rNJ-S/Jos-L-3	41	1,000	0	+	–	–
rNJ-S/Jos-L-4	41	1,600	0	+	–	EQ
rNJ-S/Jos-L-5	41	1,000	0	+	–	–

* *Antibody titration was tested using serial 4-fold dilutions starting at a serum dilution of 1:100; values represented as 1: X. DPI, day post-infection; EQ, equivocal result; IFA, immunofluorescence assay; GPC, glycoprotein precursor; Mock, historical DMEM inoculated control animals; NP, nucleoprotein; Z, matrix RING zinc-finger protein*.

## Discussion

To date, investigations into LASV reassortment have been limited to interspecies studies using LASV and another closely related old world arenavirus, MOPV (Lukashevich, 1992; Lukashevich et al., 2005) While these studies provided key support for the potential of arenavirus reassortment and introduced a novel vaccine candidate, none have specifically focused on LASV inter-species reassortment. To conduct initial investigations into intra-species reassortment of LASV, our studies focused on intra-clade reassortment between the Josiah strain, an isolate from a 1976 case of LF in Segbwema, Sierra Leone (Wulff and Johnson, 1979; Albariño et al., 2011) and a geographically and phenotypically related NJ2015 isolate, obtained from a LF case imported into USA in 2015. These 2 strains, isolated almost 4 decades apart, remain relevant to modern investigations of reassortment. Our data, along with previous studies investigating LASV sequence homology, support clustering of LASV strains based on geography as opposed to temporally (Ogbu et al., 2007; Safronetz et al., 2013; Andersen et al., 2015). Numerous Sierra Leonean LASV isolates collected between 2009 and 2013 retain remarkable homology to the 1976 Josiah isolate, meaning that Josiah-like strains currently co-circulate with NJ2015 and NJ2015-like strains.

LASV NP and Z proteins have been previously described as virulence factors *in vitro* (Hastie et al., 2012; Pythoud et al., 2012; Rodrigo et al., 2012; Jiang et al., 2013; Reynard et al., 2014; Huang et al., 2015; Xing et al., 2015). L and GPC are virulence factors for other arenaviruses, such as Pichinde and Junin, but have not been reported as virulence factors for LASV to date (Droniou-Bonzom et al, 2011; Kumar et al., 2012; McLay et al., 2013; Manning et al., 2017). *In vivo* studies investigating the contributions of individual LASV proteins to pathogenicity are fewer. Similar to previous reports of LASV strain-specific disease severity *in vivo* (Safronetz et al., 2015), none of the animals infected with rNJ2015 succumbed to disease, compared to 80% of those infected with rJosiah. When guinea pigs were infected with reassortant variants, including the rJosiah S segment alone resulted in clinical course and clinical pathology similar to those of the parental strain. Conversely, animals infected with the reassortant containing the rNJ2015 S segment and the rJosiah L segment demonstrated even milder disease than that observed in animals infected with rNJ2015, which developed clinical signs but recovered.

These observations indicate that the S segment alone is sufficient for maintaining virulence *in vivo in* this model. A similar result was observed for Junin virus reassortants where the S segment, specifically GPC, was identified as the major determinant of pathogenesis and attenuation in the guinea pig model (Seregin et al., 2015). Of note, despite non-significant differences in survival between rJosiah and rJos-S/rNJ-L, levels of some analytes differed between viruses. In particular, ALT was elevated in rJosiah, but not rJos-S/rNJ-L. Whether these differences reflect decreased hepatic damage in the reassortant is unclear, but future studies should look specifically at cellular damage when characterizing pathogenicity of reassortants and investigate whether these findings, in general, translate in additional models of disease, such as the non-human primate model (Walker et al., 1975; Jahrling et al., 1980; Jahrling and Peters, 1984).

Interestingly, S segment-specific virulence is not universal among arenavirus reassortants, with contrasting *in vivo* data reported for other species. In LCMV studies in both mice and guinea pigs, virulence was associated with the L segment (Riviere and Oldstone, 1986; Djavani et al., 1998), and in the LASV/MOPV ML29 reassortant variant the LASV S segment did not confer virulence *in vivo* (Lukashevich et al., 2005; Carrion et al., 2007; Zapata et al., 2013). The reasons for these reported differences are unclear, but in this study are likely related to the intra-clade relationship between Josiah and NJ2015 strains; rJos-S/rNJ-L may have retained virulence because the phylogenetically related NJ2015 L segment-encoded elements are more compatible with the Josiah S segment-encoded elements than those of the other arenavirus species examined previously. Taken together, our data suggest that, for LASV at least, virulence is not solely linked to the presence of one particular genome segment, and other factors like cis- and trans-acting compatibility between the segments may play a role. Given that the greater genetic distance between LASV and MOPV was enough to confer avirulence in the ML29 reassortant, it would be interesting to investigate the phenotypic differences in various inter-clade LASV reassortants of differing genetic heterogeneity.

Reassortment has been shown experimentally for several arenaviruses species, including LASV, MOPV, Junin virus, Pichinde virus, and lymphocytic choriomeningitis virus (Vezza and Bishop, 1977; Riviere and Oldstone, 1986; Djavani et al., 1998; Zhang et al., 2001; Liang et al., 2009; Seregin et al., 2015) yet little evidence exists of natural LASV reassortment. Recent large-scale sequence projects show only infrequent natural LASV reassortment: of 194 isolates, only 3 reassortant variants were reported (Andersen et al., 2015). The reason for limited reports of reassortment in nature is unclear. Possible explanations include insufficient LASV sequence data until recently, geographic barriers preventing co-circulation of LASV strains, or molecular barriers reducing the prevalence of reassortant variants. The latter would include incompatibility of cognate viral proteins and inability to recognize cis-acting elements required for vRNA synthesis or packaging on the genome segments. However, our data demonstrate cross-strain functionality of cis- and trans-acting elements between Josiah and NJ2015, supporting previous reports of LASV and MOPV cross-species functionality in minigenome assays (Kerber et al., 2011). Thus, molecular barriers do not appear to limit reassortment.

While molecular barriers do not prohibit reassortment, they may impact viral replication of reassortants in multiple ways by uncoupling protein-protein or RNA-protein interactions between S and L genome elements. These effects, termed constellation effects or segment mismatch, are probably best characterized in influenza A virus, where reassortment between the 8 genomic segments plays a major role in viral evolution (Greenbaum et al., 2012; White and Lowen, 2018). In segmented viruses, the interaction of NP and L to form the replication complex is vital to efficient vRNA synthesis. Studies have shown that inefficient interactions between influenza A virus polymerase subunits and NP can result in suboptimal activity of the replication complex (Li et al., 2008; Song et al., 2011). We similarly showed that using NP and L from alternate strains can result in reduced minigenome activity and an associated attenuated viral reassortant phenotype in rNJ-S/Jos-L. This may suggest that NJ2015 NP functionality within the replication complex is reduced compared to Josiah NP, but is offset by a higher polymerase activity of NJ2015 L. The rNJ-S/Jos-L variant uncouples this NP-L balance, resulting in the attenuated phenotype observed. The increased minigenome activity observed with Josiah NP and NJ2015 L would thus be the consequence of enhanced interactions between 2 optimal protein components of the replication complex. Furthermore, interactions between LASV L segment-encoded Z and S segment-encoded NP and GPC are important in virion assembly (Eichler et al., 2004), cellular localization (Strecker et al., 2006; Capul et al., 2007), and budding (Schlie et al., 2010). Unfortunately, lack of suitable antibodies prevented us from effectively determining levels of the L segment-encoded proteins (L and Z) expressed by the reassortant variants. However, minigenome data suggest that expression levels of these proteins may be altered compared to parental virus. Therefore, the variations in viral phenotype we observed in the reassortant viruses may be due to disruption of one or more of these processes.

Here, we demonstrate the potential for attenuation in reassortant LASV. While reassortant viruses could circulate in reservoir species, an attenuated phenotype may explain the low-frequency of detection in nature. If reassortant viruses are associated with lower incidence of severe human disease, then they may be missed as a result of sampling bias. As the majority of existing sequences are derived from isolates obtained from human cases, further studies in the *Mastomys natalensis* vector, where attenuation is advantageous for virus maintenance, could aid in identifying additional natural reassortant viruses. Importantly, our studies support the presence of an S segment-encoded virulence factor as important for disease development in this model. This work lays the foundation for future studies to characterize elements that support or disrupt protein-protein or RNA-protein interactions between heterologous S and L genome elements, and to pinpoint aspects of the S segment genome that may be targeted for therapeutic intervention.

## Author's note

The findings and conclusions in this report are those of the authors and do not necessarily represent the official position of the Centers for Disease Control and Prevention.

## Author contributions

SW, CA, and JS designed the studies; SW, FS, CA, MK, JC-M, LW, AC, and JS performed the experiments; SW, FS, CA, MK, and JS analyzed and interpreted the data; SW and JS wrote the manuscript; JK, SN, and CS provided resources and supervision. All authors contributed to manuscript revision, and have read and approved the submitted version.

### Conflict of interest statement

The authors declare that the research was conducted in the absence of any commercial or financial relationships that could be construed as a potential conflict of interest.

## Supplementary material

Supplementary Figure 1 Minigenome activity of LASV strains NJ2015 and Josiah with cognate and non-cognate cis-acting factors. Minigenomes segments were created to quantify the transcriptional strengths of the NCRs located on the S and L genome segments of Josiah and NJ2015 strains of LASV. Represented here are the ZsG fluorescence values (average background ZsG fluorescence of mock transfected Huh7 cells is removed from all values) and the gLuc luminescence values for all minigenomes segments, representing the 5′ NCR and the 3′ NCR respectively. All minigenome reactions were performed in conjunction with a negative control (no polymerase, just pCAGGS) to assess background levels of minigenome activity in the absence of viral transcriptional machinery. + represent the combination of cis-acting factors transfected. NP, nucleoprotein; L, polymerase; pCAGGS, empty plasmid.

Supplementary Figure 2 Western blot and qRT-PCR analysis of innate immune responses in A549 cells. **(A)** Antiviral cellular protein expression levels in A549 cells infected with the 4 recombinant LASV strains at MOI 7. Protein lysates were harvested at 24 hpi and probed for STAT1, pSTAT1, and ISG15. A549 cells infected with Rift Valley fever virus lacking NSs and NSm (RVFVΔΔ; Bird et al., 2008) at MOI 1 were used as a positive control. Uninfected A549 were used as mock (Ø), and tubulin was used as loading control. **(B)** qRT-PCR analysis of IFN-β, IFN-λ, ISG56, CCL5, and IL-6 transcript levels in A549 cells infected with the 4 recombinant LASV strains at MOI 7, with RNA harvested at 24 hpi. Cells infected with RVFVΔΔ at MOI 1 were used as a positive control and uninfected A549 used as mock (Ø). Values are represented as fold induction over mock infected cells, with lines representing statistical significance, one-way ANOVA with Dunnett's multiple comparison test (^*^*p* < 0.05; ^**^*p* < 0.01; ^***^*p* < 0.001; ^****^*p* < 0.0001; ns = not significant). Also shown are LASV S segment viral RNA levels in the infected cells, with values representing fold difference compare to rJosiah infected cells.

Supplementary Table 1 Details of the LASV and MOPV sequences used for phylogenetic analysis.
