# Supplementary material for: The S Genome Segment Is Sufficient to Maintain Pathogenicity in Intra-Clade Lassa Virus Reassortants in a Guinea Pig Model
Source: Front Cell Infect Microbiol. 2018 Jul 11;8:240. doi: 10.3389/fcimb.2018.00240 (PMC6050391; doi:10.3389/fcimb.2018.00240)
Supplement: Supplementary Table 1 — Details of the LASV and MOPV sequences used for phylogenetic analysis. [file Table_1.docx]

**Supplementary Table 1**: Details of the LASV and MOPV sequences used for phylogenetic analysis.

| Clade | Country | Year Isolated | Strain Name | Genbank# S | Genbank# L | Reference |
| --- | --- | --- | --- | --- | --- | --- |
| 1 | Nigeria | 1969 | Pinneo | KM822128.1 | KM822127.1 | (Andersen et al., 2015) |
| 2 | Nigeria | 1974 | 810801-NIG-1974 | MG812681 | MG812680 | N/A |
| 2 | Nigeria | 1989 | 806593-NIG-1989 | KU978811.1 | KU978812.1 | N/A |
| 2 | Nigeria | 2008 | Nig08-04 | GU481068.1 | GU481069.1 | (Ehichioya et al., 2010) |
| 2 | Nigeria | 2008 | Nig08-A47 | GU481078.1 | GU481079.1 | (Ehichioya et al., 2011) |
| 2 | Nigeria | 2009 | LASV035-NIG-2009 | KM822004 | KM822003 | (Andersen et al., 2015) |
| 2 | Nigeria | 2010 | LASV221-NIG-2010 | KM822022 | KM822021 | (Andersen et al., 2015) |
| 2 | Nigeria | 2011 | ISTH0009-NIG-2011 | KM821912.1 | KM821911.1 | (Andersen et al., 2015) |
| 2 | Nigeria | 2012 | ISTH1121-NIG-2012 | KM821945.1 | KM821944.1 | (Andersen et al., 2015) |
| 3 | Nigeria | 1981 | 807876-NIG-1981 | MG812635 | MG812634 | N/A |
| 3 | Nigeria | 2008 | Nig08-A18 | GU481070.1 | GU481071.1 | (Ehichioya et al., 2010) |
| 3 | Nigeria | 2008 | Nig08-A19 | GU481072.1 | GU481073.1 | (Ehichioya et al., 2010) |
| 4 | Liberia | 1972 | 803204-LBR-1972 | MG812648 | MG812649 | N/A |
| 4 | Sierra Leone | 1975 | 803209-SLE-1975 | MG812647 | MG812646 | N/A |
| 4 | Sierra Leone | 1976 | Josiah | AY628203.1 | AY628202.1 | N/A |
| 4 | Sierra Leone | 1977 | 806827-SLE-1977 | MG812639 | MG812638 | N/A |
| 4 | Sierra Leone | 1979 | 806568-SLE-1979 | MG812643 | MG812642 | N/A |
| 4 | Liberia | 1980 | Z148 | AY628205.1 | AY628204.1 | N/A |
| 4 | Liberia | 1981 | 806829-LBR-1981 | MG812636 | MG812637 | N/A |
| 4 | Guinea | 1981 | Macenta | AY628201.1 | AY628200.1 | N/A |
| 4 | Guinea | 1996 | Guinea Faranah | KU978807.1 | KU978808.1 | N/A |
| 4 | Sierra Leone | 2000 | NL | AY179173.1 | AY179172.1 | (Schmitz et al., 2002) |
| 4 | Sierra Leone | 2009 | G502-SLE-2009 | KM821773.1 | KM821772.1 | (Andersen et al., 2015) |
| 4 | Sierra Leone | 2009 | G692-SLE-2009 | KM821783.1 | KM821782.1 | (Andersen et al., 2015) |
| 4 | Sierra Leone | 2009 | L395-SLE-2009 | KM822115 | KM822114 | (Andersen et al., 2015) |
| 4 | Liberia | 2010 | 811606-LBR-USA-2010 | MG812679 | MG812678 | N/A |
| 4 | Sierra Leone | 2010 | G1180-SLE-2010 | KM821794.1 | KM821793.1 | (Andersen et al., 2015) |
| 4 | Sierra Leone | 2011 | G1442-SLE-2011 | KM821800.1 | KM821799.1 | (Andersen et al., 2015) |
| 4 | Sierra Leone | 2011 | G2222-SLE-2011 | KM821832.1 | KM821831.1 | (Andersen et al., 2015) |
| 4 | Sierra Leone | 2012 | G2259-SLE-2012 | KM821835.1 | KM821834.1 | (Andersen et al., 2015) |
| 4 | Sierra Leone | 2012 | LM779-SLE-2012 | KM822126 | KM822125 | (Andersen et al., 2015) |
| 4 | Sierra Leone | 2013 | G3010-SLE-2013 | KM821882.1 | KM821881.1 | (Andersen et al., 2015) |
| 4 | Liberia | 2014 | 812337-LBR-USA-2014 | MG812658 | MG812659 | N/A |
| 4 | Liberia | 2015 | 812673-LBR-USA-2015 | MG812650 | MG812651 | N/A |
| 5 | Ghana, CI, BF | 2000 | AV | AF246121.2 | AY179171.1 | (Günther et al., 2000) |
| 5 | Mali | 2012 | Bamba-R114 | KF478766.1 | KF478761.1 | (Safronetz et al., 2013) |
| 5 | Mali | 2012 | Komina-R16 | KF478767.1 | KF478760.1 | (Safronetz et al., 2013) |
| 5 | Mali | 2012 | Ouoma-R123 | KF478768.1 | KF478764.1 | (Safronetz et al., 2013) |
| 5 | Mali | 2012 | Soromba-R | KF478765.1 | KF478762.1 | (Safronetz et al., 2013) |
| 5 | Mali | 2012 | Soromba-R30 | KF478769.1 | KF478763.1 | (Safronetz et al., 2013) |
| 6 | Togo | 2016 | Togo/2016/7082 | KU961971.1 | KU961972.2 | N/A |
| N/A | Mozambique | 1972 | Mopeia AN20410 | AY772170.1 | AY772169.1 | (Wulff et al., 1977) |
| N/A | Mozambique | unknown | Mopeia Mozambique | DQ328874.1 | DQ328875.1 | (Emonet et al., 2006) |
| N/A | N/A | N/A | rJosiah | HQ688673.1 | HQ688675.1 | (Albariño et al., 2011) |

References included where applicable. CI, Côte d’Ivoire; BF, Burkina Faso; N/A, not applicable.

**REFERENCES:**

Albariño, C. G., Bird, B. H., Chakrabarti, A. K., Dodd, K. A., Erickson, B. R., and Nichol, S. T. (2011). Efficient rescue of recombinant Lassa virus reveals the influence of S segment noncoding regions on virus replication and virulence. *J. Virol.* 85, 4020–4024. doi: 10.1128/JVI.02556-10

Andersen, K. G., Shapiro, B. J., Matranga, C. B., Sealfon, R., Lin, A. E., Moses, L. M., et al. (2015). Clinical sequencing uncovers origins and evolution of Lassa virus. *Cell* 162, 738–750. doi: 10.1016/j.cell.2015.07.020

Ehichioya, D. U., Hass, M., Becker-Ziaja, B., Ehimuan, J., Asogun, D. A., Fichet-Calvet, E., et al. (2011). Current molecular epidemiology of Lassa virus in Nigeria. *J. Clin. Microbiol*. 49, 1157–1161. doi: 10.1128/JCM.01891-10

Ehichioya, D. U., Hass, M., Olschläger, S., Becker-Ziaja, B., Onyebuchi Chukwu, C. O., Coker, J., et al. (2010). Lassa fever, Nigeria, 2005-2008. Emerg. Infect. Dis. 16, 1040–1041. doi: 10.3201/eid1606.100080

Emonet, S., Lemasson, J.-J., Gonzalez, J.-P., de Lamballerie, X., and Charrel, R. N. (2006). Phylogeny and evolution of old world arenaviruses. Virology 350, 251–257. doi: 10.1016/j.virol.2006.01.026

Günther, S., Emmerich, P., Laue, T., Kühle, O., Asper, M., Jung, A., et al. (2000). Imported lassa fever in Germany: molecular characterization of a new lassa virus strain. Emerg. Infect. Dis. 6, 466–476. doi: 10.3201/eid0605.000504

Safronetz, D., Sogoba, N., Lopez, J. E., Maiga, O., Dahlstrom, E., Zivcec, M., et al. (2013). Geographic distribution and genetic characterization of Lassa virus in sub-Saharan Mali. *PLoS Negl. Trop. Dis*. 7:e2582. doi: 10.1371/journal.pntd.0002582

Schmitz, H., Köhler, B., Laue, T., Drosten, C., Veldkamp, P. J., Günther, S., et al. (2002). Monitoring of clinical and laboratory data in two cases of imported Lassa fever. Microbes Infect. 4, 43–50.

Wulff, H., McIntosh, B. M., Hamner, D. B., and Johnson, K. M. (1977). Isolation of an arenavirus closely related to Lassa virus from Mastomys natalensis in south-east Africa. Bull. World Health Organ. 55, 441–444.
